# The Routine Utilization of Procedural Pain Management for Pediatric Lumbar Punctures: Are We There Yet?

**DOI:** 10.4021/jocmr584w

**Published:** 2011-07-26

**Authors:** Julie Gorchynski, Thomas McLaughlin

**Affiliations:** a Department of Emergency Medicine, JPS Health Network, Fort Worth, TX, USA; b Department of Emergency Medicine, Christus Spohn Memorial Hospital, Corpus Christi, TX, USA

## Abstract

**Background:**

The objective of this study was to assess the utilization of local anesthetics by emergency physicians (EP) and pediatric physicians (PP) who performed a lumbar puncture (LP) in pediatric patients from birth to 24 months of age.

**Methods:**

We conducted a prospective study of children that received an LP at a university tertiary referring hospital. A convenience sample included children from birth to 24 months that received an LP for suspected meningitis in the ED or pediatric units during a one-year period. Physicians performing the LP were blinded to the objectives of the study. Data was collected using a standardized procedure form developed for this study.

**Results:**

Three hundred nine LPs were performed during the study period. Excluded patients consisted of 29 subjects who underwent moderate procedural sedation and 57 subjects that had incomplete procedural data forms. From our sample population of 223 subjects, 146 subjects received a local anesthetic prior to the LP. One hundred twenty six subjects received 1% lidocaine, 20 subjects received EMLA cream (with one subject that received both 1% lidocaine and EMLA), while 77 received no pre-procedural local anesthetic. The use of local anesthetics differed greatly with the age of the patient. Pre-procedural local anesthetics were administered in 65 of 120 subjects less than 12 months of age and in 81 of 82 patients 12 to 24 months of age. Interestingly, the neonatal subject population did not receive any procedural anesthetic by EP or PP. PP and EP differed in the type of local anesthetic utilized prior to performing a LP. EP exclusively used 1% lidocaine while PP preferentially administered EMLA. A subset analysis demonstrated that only PP utilized moderate sedation (Midazolam and Fentanyl) in 41/309 (13%) of the study population.

**Conclusions:**

This is the first study to demonstrate that EPs and PPs differ in their preference in the use of local anesthetics prior to LP and that procedural anesthetic is not universal within this pediatric age group and that utilization of a local anesthetic varies by patient age, with younger children less likely to receive a local anesthetic.

**Keywords:**

Local anesthetic; Lumbar puncture; Emergency physician; Pediatric physician

## Introduction

Lumbar punctures (LP) are frequently performed on emergency and hospitalized pediatric patients to diagnose meningitis. To reduce pain, analgesics are commonly utilized prior to LPs in adult and older pediatric patients. Younger pediatric patients, however, have historically undergone invasive medical procedures including LPs without the use of analgesics. Although the American College of Emergency Physicians (ACEP) in 1997 and the American Academy of Pediatrics (AAP) in 2001 have advocated for the routine utilization of pain management in all pediatric patients, procedures continue to be performed on pediatric patients without analgesia [1-4]. The lack of procedural analgesia may have long term consequences as recent studies indicate that painful experiences during neonatal and infant development may alter subsequent pain pathway development and decrease pain tolerance [5, 6]. The objective of this study was to assess the utilization of local anesthetics in pediatric LPs and to identify the type of local anesthetic used by emergency physicians (EP) and pediatrics physicians (PP) who performed an LP for suspected meningitis in pediatric patients from birth to 24 months of age.

## Materials and Methods

A prospective study was conducted at a university tertiary hospital with an annual census of 45,000 patients. The study was conducted over a one-year period and included all patients from birth to 24 months of age who underwent an LP in the emergency department (ED) or the inpatient pediatric unit.

A procedural data form specific for this study was completed by both EPs and PPs after the LP was performed. Incomplete procedural data forms were completed by data abstraction from the medical charts. Data was collected for demographics, date of LP, cerebral spinal fluid (CSF) cell count, CSF culture results, type of analgesics utilized, the department in which the LP was performed, the medical specialty of the physician and the number of attempts performed during the LP.

The hospital laboratory provided the medical record number of all CSF specimens submitted to the laboratory for analysis to cross check for all LPs performed during the study period. All LPs performed in the ED were either performed or supervised by an EP and all LPs performed on the inpatient pediatric units were performed or supervised by a pediatrician. To assess the accuracy of data abstraction ten medical records were randomly chosen and reviewed by a second EP yielding an assessment of inter-rater reliability. The institutional review board approved the study. Data were analyzed using STATA (version 7.0, Stata Corp., College Station, Texas).

## Results

**Figure 1.**
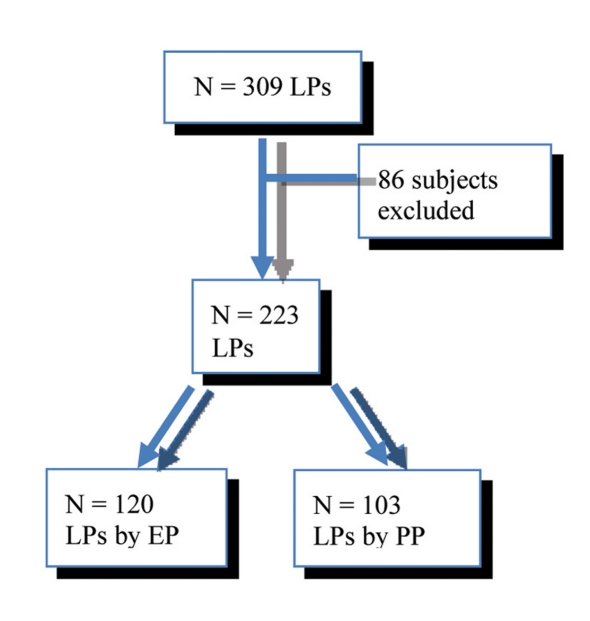
Flow diagram of pediatric LP subjects.

A total of 309 LPs were performed during the study period. Eighty-six subjects were excluded due to the use of procedural sedation (41) or incomplete data (45) (Fig. 1). The sample population of 223 subjects consisted of 101 males and 122 females with an average age of 10.5 months. Totally, 146/223 (66%) of the subjects received some type of local anesthetic prior to the LP; 126 subjects (57%) received 1% lidocaine, 20 subjects (9%) received EMLA and 77 subjects (35%) received no procedural anesthetic (Table 1).

**Table 1:** Type of Local Anesthetic Utilized in Pediatric LPs

N = 223	n	P (%)	95% CI
1% lidocaine	126	57	49.5 - 62.5
EMLA	20	9	5.2 - 12.6
EMLA or 1% lidocaine	146	66	58.7 - 71.3
None	77	35	27.8 - 40.2
Other*	15	7	3.4 - 10.0

* Tylenol or NSAID or BDZ or Narcotic

The majority of toddlers received some form of local anesthetic while infants received a local anesthetic only half the time. Interestingly, the neonatal subject population did not receive any procedural anesthetic by EP or PP (Table 2, 3).

**Table 2:** Local Anesthetic Utilized in Pediatric LPs Stratified by Age

Age	N = 223	n	P (%)	95% CI
Birth to 30 days	21	0	0	0 - 0.15
> 1 month to 12 months	120	65	54	45.1 - 62.9
> 12 months to 24 months	82	81	99	95.0 - 101

**Table 3:** Type of Local Anesthetic Utilized in Pediatric LPs Stratified by Age

Age	N	1% lidocaine		EMLA		Both	
		n	P (%)	n	P (%)	n	P (%)
Neonate	21	0	0	0	0	0	0
Infant	120	75	60	3	3	3	3
Toddler	82	51	62	17	21	2	2

A subset analysis resulted in 41/309 (13%) of the subjects that received conscious sedation (fentanyl and midazolam). These subjects were between 14 months to 24 months of age. In this study, 187/309 (61%) of the subjects received either a local anesthetic or conscious sedation.

EPs and PPs differed in the type of local anesthetic utilized. EPs exclusively used 1% lidocaine while pediatricians also used EMLA (Table 4). Only PPs utilized conscious sedation prior to LP within this study population.

**Table 4:** Type of Local Anesthetic Utilized in Pediatric LPs Stratified by Physician Specialty

LP	N = 223	n = 146	P (%)	1% lidocaine		EMLA		Both	
				**n**	**P (%)**	**n**	**P (%)**	**n**	**P (%)**
EP	N = 120	82	68	82	68	0	0	0	0
PP	N = 103	64	62	44	43	20	19	5	4

The Kappa (k) value for inter-rater reliability in data abstraction was 0.65.

## Discussion

This study demonstrated that the utilization of local anesthetic prior to LP in pediatric subjects from birth to 24 months of age is highly variable with children less than 12 months of age less likely to receive local procedural anesthetics than children over 12 months of age. This data supports previous studies that found younger patients are less likely to receive local anesthetics than older pediatric patients [1, 4, 6-8]. This study demonstrated that the neonatal population did not receive any procedural analgesia by either EP or PP. This is unfortunate since there is not any literature supporting the disuse of procedural anesthetics in this age group. On the contrary, there is an abundance of literature that supports and recommends procedural pain management among neonates [3].

This study identified a difference between PPs and EPs in the type of procedural anesthetic used. EM physicians preferentially utilized 1% lidocaine while pediatricians more commonly utilized EMLA or conscious sedation. This difference most likely reflects each specialty's respective time pressures, practice environment and training. EMLA requires approximately 60 minutes to penetrate the dermis for effective analgesia, which makes its use in the ED impractical because of the urgency to obtain CSF and initiate antibiotics in cases of suspected meningitis.

Until the late 1990's the use of procedural analgesia for LP was not commonly utilized or emphasized among physicians in EM or pediatrics. In 1993, Quinn reported a difference between EM physicians and pediatricians in their frequency of use of local anesthetic prior to LPs. Quinn reported that only 4.5% of children in a pediatric ED staffed by pediatricians received local anesthetics prior to an LP compared to 93% of children receiving a local anesthetic prior to an LP in a general ED staffed by EM physicians [7]. Although our study demonstrated an improved rate of utilization of local anesthetics prior to LP over that found by Quinn, pediatric procedures continue to be performed without procedural anesthesia [2, 9]. This is unfortunate because numerous studies have demonstrated that infants and children have a well developed ability to sense pain and that painful events can have an extended adverse effect upon development of pain pathways and responses to future painful events [5].

Frequent reasons described in the literature for not using procedural anesthetics in the pediatric population include that the LP may require a second unnecessary stick, the wheal formed by the local anesthetic obscures landmarks, the injection of the lidocaine is as painful as the LP itself, and that giving a local anesthetic involves a second injection, which takes as much time as the LP itself [7]. Interestingly, a majority of the physicians who cited the above reasons felt that these factors were age dependant and applied preferentially to infants [7]. Separate studies conducted by Pinheiro, Corracio, and Baxter demonstrated that the use of local anesthetics does not alter the success of outcome of pediatric LP [9, 10].

Another concern regarding the hesitancy to utilize local anesthetics may be their possible toxic effects. However, 1% idocaine in doses that do not exceed 4 mg/kg, or a single application of EMLA have been shown to be safe in the pediatric population [3, 11].

Limitations to this study include the unknown neurological status and other pertinent abnormal physical characteristics of the subject, which may have affected the physicians' decision regarding the use of local anesthetics. The results of this study may not be representative of other institutions since it is a referral center and not a children's hospital that encounters a higher number of pediatric LPs.

In conclusion, the under utilization of procedural analgesia continues to occur despite ACEP and AAP policy statements and numerous studies demonstrating their safe use in pediatric patients. All pediatric patients, regardless of age should receive procedural pain management.
